# Transcript-Wide Identification and Characterization of the BBX Gene Family in *Trichosanthes kirilowii* and Its Potential Roles in Development and Abiotic Stress

**DOI:** 10.3390/plants14060975

**Published:** 2025-03-20

**Authors:** Weiwen Li, Rui Xiong, Zhuannan Chu, Xingxing Peng, Guangsheng Cui, Ling Dong

**Affiliations:** 1 Key Laboratory of Horticultural Crop Germplasm Innovation and Utilization (Co-Construction by Ministry and Province), Institute of Horticulture, Anhui Academy of Agricultural Sciences, Hefei 230001, China; m15255173358@163.com (R.X.); chuzhnan1988@163.com (Z.C.); 15212447331@163.com (X.P.); cuigs8@163.com (G.C.); 2 Anhui Provincial Key Laboratory for Germplasm Resources Creation and High-Efficiency Cultivation of Horticultural Crops, Hefei 230001, China

**Keywords:** phylogenetic analysis, expression patterns, salt stress, drought stress

## Abstract

*The B-box (BBX) protein* has an impact on flowering physiology, photomorphogenesis, shade effects, and responses to both biotic and abiotic stresses. Although recent research described the BBX gene family in numerous plants, knowledge of the BBX gene in *Trichosanthes kirilowii* was sparse. In this study, we identified a total of 25 *TkBBX* genes, and phylogenetic analysis showed that these genes were divided into five subfamilies. Analyses of gene structure and motifs for each group found relative conservation. Ka/Ks values showed that most *TkBBX* genes have undergone negative selection. qRT-PCR analyses revealed that *TkBBX1*, *TkBB4*, *TkBBX5*, *TkBBX7*, *TkBBX15*, *TkBBX16*, *TkBBX17*, *TkBBX19*, and *TkBBX21* genes respond to salt and drought treatment. Furthermore, we cloned *TkBBX7* and *TkBBX17* genes and performed a subcellular localization experiment, which revealed that these two genes were both located in the nucleus. Transgenic yeast experiments demonstrated that *TkBBX7* and *TkBBX17* enhanced yeast tolerance to both salt and drought stresses. These findings provide a theoretical foundation for further investigation on the functions of *TkBBX* genes in *Trichosanthes kirilowii*.

## 1. Introduction

The zinc finger protein (ZEP) transcription factor family is one of the largest plant transcription factor families and plays an important role in plant growth, development, and response to abiotic stress [1,2,3]. The B-box (BBX) protein family belongs to a subfamily of ZEP and contains one or two B-box domains, and some family factors also have CONSTANS, CO-like, and TOC1 (CCT) domains [4]. The B-box motif contains about 40 amino acid residues, which can be divided into two types, B-box 1 and B-box 2, based on the consistency and difference of amino acids and zinc ion binding sites. Both of them are highly conserved and mainly play roles in protein interaction and transcriptional regulation. The CCT domain, consisting of 42 to 43 amino acid residues, is highly conserved and mainly involved in protein intranuclear transport and transcriptional regulation [1].

BBX genes play an important role in plant growth and development [5], including flowering physiology, photomorphogenesis, and shade effects. CONSTANS (CO) in *Arabidopsis thaliana* was the first BBX protein discovered in plants [6]. *AtBBX6* and AtBBX24 can promote early flowering [7,8], while *AtBBX4*, *AtBBX7*, and *AtBBX32* are negative regulators of flower formation [9,10]. In chrysanthemums, overexpression of CmBBX8 promoted flowering under both long- and short-day conditions. CmFTL1 induced blooming under long-day circumstances, while CmBBX8 was proved to promote flowering by binding to the CmFTL1 promoter’s transient acting element (CCACA) [11]. *OsCOL4*, *OsBBX14*, and *OsCOL9* in rice delay plant heading through the Ehd1 pathway [12,13]. BBX can regulate some biological processes such as the growth of the embryo axis, the growth of lateral roots, and the spreading of seed leaves [14]. In *Arabidopsis thaliana*, *AtBBX4*, *AtBBX20*, *AtBBX21*, and *AtBBX22* can promote photomorphogenesis [15,16,17,18,19], while *AtBBX18*, *AtBBX19*, *AtBBX24*, *AtBBX25*, and *AtBBX32* can inhibit it [20,21,22,23,24]. Proteins in apples with the same structural domain as *AtBBX1*, *MdBBX22* [25], and *MdBBX20* [26] were shown to positively regulate light-induced anthocyanin accumulation through the light input pathway. When the plant is in a shaded environment, the BBX protein can mediate cell elongation, thereby increasing the height of the plant [27]. In shaded environments, *AtBBX19*, *AtBBX21*, and *AtBBX22* were proved to inhibit cell elongation, while AtBBX18, *AtBBX24*, and *AtBBX25* can promote cell elongation, thereby causing hypocotyl elongation [20,28]. *MdBBX37* can inhibit *MdMYB1* and *MdMYB9* binds to target genes, thereby inhibiting anthocyanin biosynthesis and hypocotyl elongation [29]. *MdPIF7* interacted with *MdBBX23*, reducing its transcriptional control on *MdHY5*, which regulated anthocyanin production and hypocotyl development [30].

The BBX proteins are also involved in the hormone signal pathway and abiotic stress response [31]. In some plants, BBX was reported to be involved in the anthocyanin synthesis pathway. HY5 promoted the expression of *CHS*, *CHI*, *F3H*, *F3`H*, and *DFR* genes during proanthocyanidin production, *AtBBX21* can promote its expression through interaction with HY5 [28], nevertheless *AtBBX24* and *AtBBX25* inhibit the expression of HY5 [20]. In apple, a BBX protein, MdCOL11, interacted with *MdHY5* to increase anthocyanin accumulation and pigmentation in the peel [32]. *PpBBX18* and *PpBBX21* antagonistically regulate anthocyanin biosynthesis in pear fruit through competitive binding with *PpHY5* [33]. BBX plays certain roles in response to salt, drought, and heat stress [1]. For example, the overexpression of *AtBBX24* increased the salt tolerance of plants [34]. The overexpression of *MdBBX10* in *Arabidopsis thaliana* significantly enhanced tolerance to salt and drought stress, and the germination rate and root length were higher than those of wild type plants [35]. Overexpression of *Ginkgo biloba* L. *BBX25* in poplar trees increased salt tolerance [36], while overexpression of *Chimonanthus praecox* L. *CpBBX19* in Arabidopsis also increased plant tolerance to salt stress [37]. The *CmBBX22* and *AtBBX22* genes were homologous, and *CmBBX22* in transgenic Arabidopsis provided a drought resistant phenotype [38]. The Arabidopsis bbx18 mutant has higher heat resistance, whereas the *AtBBX18* overexpression strain has lower heat resistance [39].

BBXs have been identified in Arabidopsis, grape, rice, apple, tomato, and pear, but not in *Trichosanthes kirilowii* Maxim. This plant belongs to the Cucurbitaceae family, and its root, peel, and seeds are all used as medicine. Due to its growing application in the therapeutic management of cardiovascular disease and cancer, *T. kirilowii* was widely cultivated in Anhui, Hebei, Shandong, Henan, and Jiangsu provinces. In the context of global climate change and land degradation, highly resistant varieties are urgently needed in daily production. Furthermore, since *T. kirilowii* is a dioecous plant, the flowering times of female and male flowers do not coincide. Studying genes that can regulate the flowering period, making the flowering time of female and male flowers consistent, can increase the yield. *T. kirilowii*, as an octaploid plant, does not have a published genome, and gene mining can only rely on transcriptome data. In this study, we analyzed the evolution of TkBBX, the expression levels of TkBBX during flowering and salt and drought treatments and screened genes for preliminary functional validation in yeast. These results can provide a reference for regulating the flowering period and cultivating new stress-resistant varieties of *T. kirilowii*.

## 2. Results

### 2.1. Identification of TkBBX Genes

According to the AtBBX Pfam number (PF00643), a total of 25 TkBBX genes named TkBBX1 to TkBBX25 were identified from the transcriptome data. Table 1 includes basic information on *T. kirilowii* BBX genes such as open reading frame (ORF) length, protein length, isoelectric point (PI), molecular weight (MW), and subcellular localization. The TkBBX length range varies from 228 bp (TkBBX22) to 1323 bp (TkBBX25) and MW from 8068.35 Da (TkBBX22) to 49,928 Da (TkBBX25). These genes encode proteins with an average size of 282 aa and a size range from 75 to 440 aa. The PI values of TkBBX genes range from 4.2 (TkBBX17) to 10.42 (TkBBX14). In terms of subcellular localization, all TkBBX genes were predicted to be located in the nucleus.

### 2.2. Phylogenetic and Classification Analysis of TkBBX Genes

**Figure 1:**
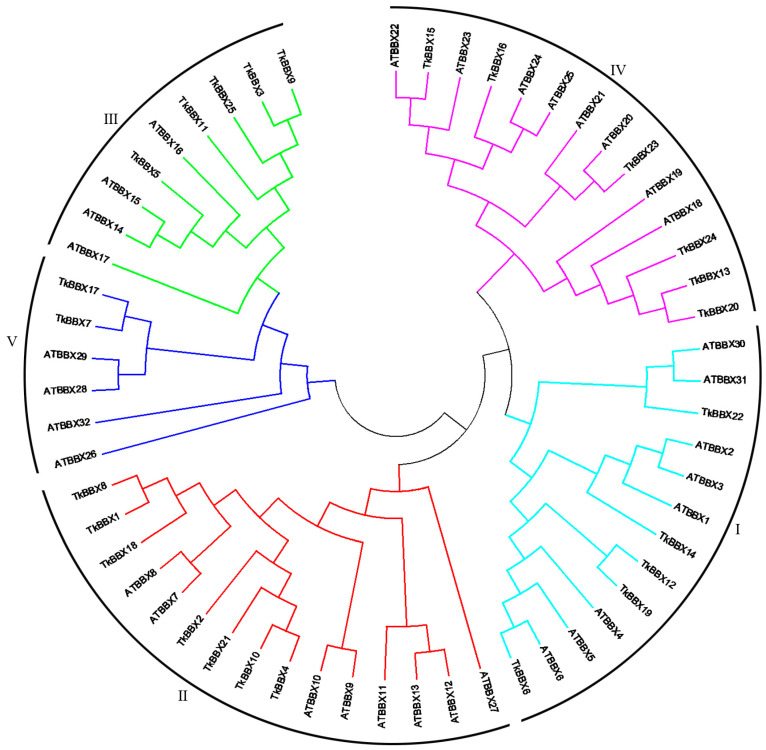
Phylogenetic tree of full-length *BBX* genes from *Trichosanthes kirilowii* and *Arabidopsis thaliana*.

To investigate the relationship and classification of BBX members in *T. kirilowii*, an unrooted neighbor-joining (NJ) phylogenetic tree of 25 TkBBX and 32 AtBBX genes was constructed (Figure 1). According to the classfication of *A. thaliana*, TkBBXs were divided into five subfamilies (I–V) with 4 (TkBBX6, 12, 14, 19), 7 (TkBBX1, 2, 4, 8, 10, 18, 21), 5 (TkBBX3, 5, 9, 11, 25), 7 (TkBBX13, 15, 16, 20, 22, 23, 24), and 2 (TkBBX7, 17) members, respectively.

The tree was constructed using the neighbor-joining (NJ) method with MEGA 11.0 based on *BBX* sequences from *Trichosanthes kirilowii* and Arabidopsis thaliana.

### 2.3. Conserved Motif and Selective Pressure Analysis of TkBBX Genes

**Figure 2:**
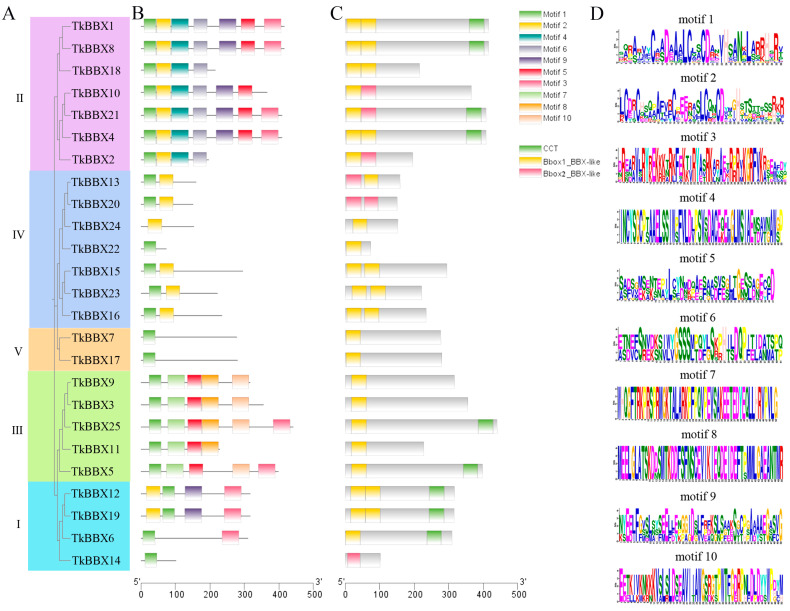
Domain, motif compositions, and distribution of conserved motifs of TkBBX proteins. (**A**): Phylogenetic tree of the TkBBX family. Colors represent the different groups. (**B**): Conserved motif analysis of TkBBX within each group. Different colored boxes represent different motifs. (**C**): Domain analysis of TkBBX within each group. (**D**): Conserved amino acid sequences and the length of each motif.

Motif analysis of 25 TkBBX genes was performed using Multiple Expectation Maximization for Motif Elicitation (MEME) online tools to investigate the conserved domain characteristics (Figure 2). As shown in Figure 2, all TkBBX genes contain at least one B-box (motif 1/motif 2). The motifs of genes which belong to the same subfamily were almost identical. For instance, motifs 1, 2, 4, and 6 appear in all genes of subfamily II, while most genes in subfamily III contained motifs 1, 5, 7, and 10.

Homologous pairs of BBX genes between *T. kirilowii* and *A. thaliana* were identified and are listed in Table 2. Ten paralogous pairs in *T. kirilowii* and nine orthologous pairs between *T. kirilowii* and *A. thaliana* were identified. To further comprehend Darwinian evolutionary selection in the TkBBX gene family, we estimated non-synonymous substitution (Ka), the synonymous substitution rate (Ks), and Ka/Ks value combinations (Table 2). The results showed that all pairs had Ka/Ks values less than 1, which indicated that all TkBBX genes underwent strong purification selection.

### 2.4. Expression Analyses of TkBBX Genes During Flowering Stage

**Figure 3:**
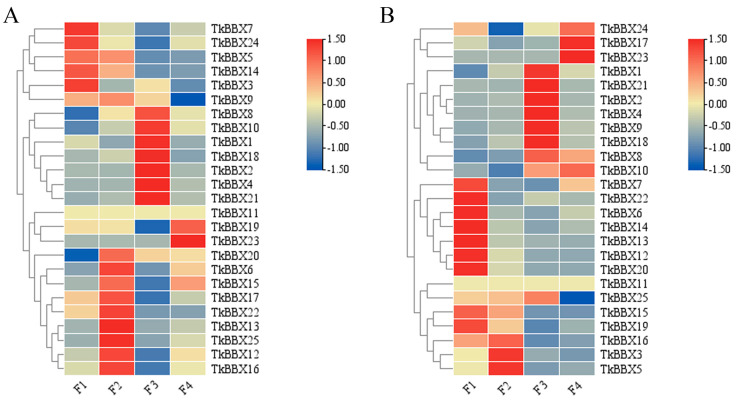
Heat map of *TkBBX* genes at different flowering stages based on transcriptome data. (**A**): Female flower; (**B**): Male flower; F1: buds; F2: preliminary bloom; F3: full bloom; F4: fade bloom.

BBX transcript factors play roles in plant growth and have an impact on flowering physiology. To analyze BBX gene expression changes during flowering, the fragments per kilobase of exon model per million mapped reads (FPKM) of 25 TkBBX genes were counted and are shown in Figure 3. The expression changes of female and male flowers at different flowering stages were compared. For female flowers, seventeen genes were differentially expressed genes (DEGs). The expression levels of TkBBX5, TkBBX7, TkBBX9, TkBBX12, and TkBBX17 were down-regulated, and the expression levels of TkBBX1, TkBBX4, TkBBX8, TkBBX10, and TkBBX21 were highest at full bloom, while expression levels of TkBBX15 and TkBBX16 were highest at preliminary bloom (Figure 3A). For male flowers, seventeen genes were DEGs. TkBBX6, TkBBX7, TkBBX12, TkBBX13, TkBBX15, and TkBBX19 were down−regulated, and TkBBX16’s expression level was highest at preliminary bloom, while TkBBX1, TkBBX4, TkBBX8, TkBBX10, and TkBBX21 were highest at full bloom (Figure 3B).

### 2.5. TkBBX Expression Pattern Responds to Salt and Drought Stresses

**Figure 4:**
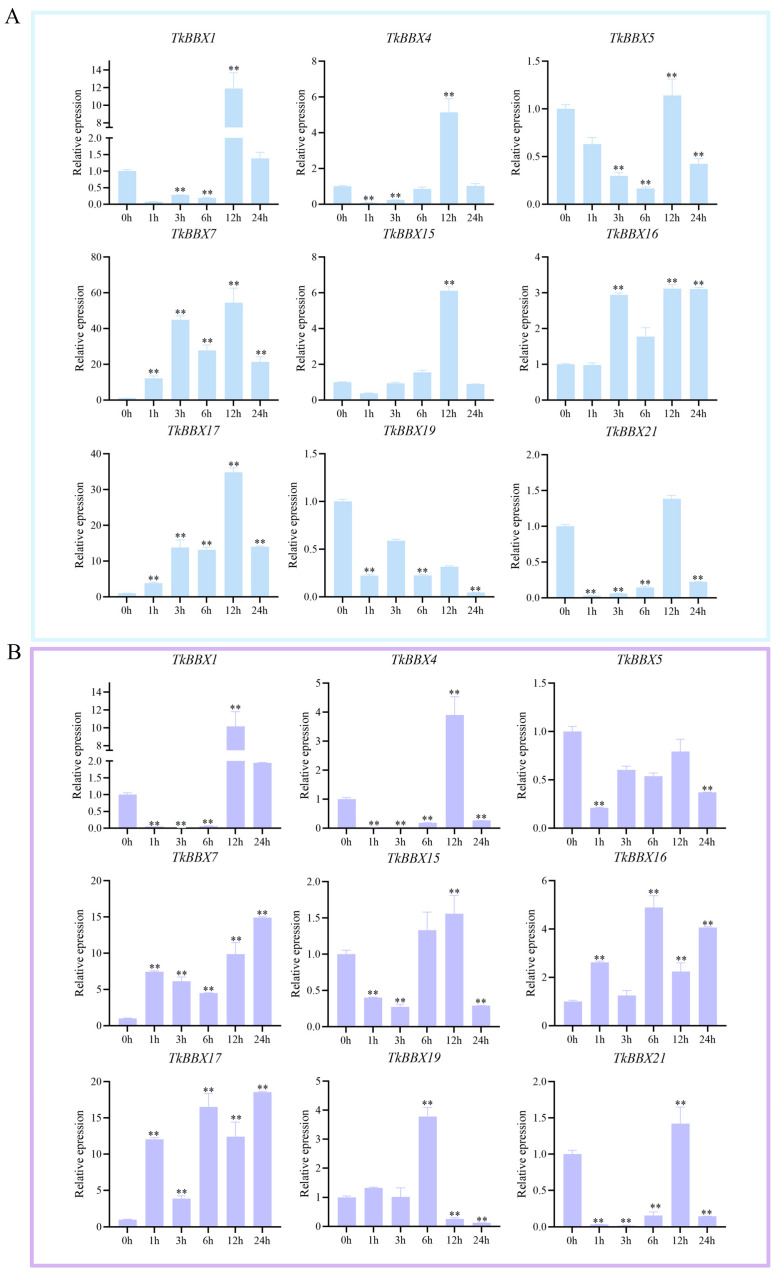
Expression analysis of 9 TkBBX genes under salt and drought treatments by qRT-PCR. (**A**): Nine *TkBBXs*’ expression levels in seedlings under 300 mM NaCl treatments. (**B**): Nine *TkBBXs*’ expression levels in seedlings under 20% PEG 6000 treatments. Error bars indicate standard deviations among three independent biological replications. **: *p*-value < 0.01.

Previous studies reported that BBX transcription factor families were involved in plant stress responses [1]. Based on the transcriptome results, nine TkBBX genes were selected to investigate their response to salt and drought stress. Under NaCl treatment (Figure 4A), the expression levels of TkBBX1, TkBBX4, TkBBX5, TkBBX15, and TkBBX21 were highest at 12 h, those of TkBBX7, TkBBX16, and TkBBX17 exhibited a continuous increase, while TkBBX19 showed a continuous decrease. Among them, the expression levels of TkBBX7 and TkBBX17 genes were strongly up-regulated at 12 h (more than 50- and 30-fold, respectively). Under PEG treatment (Figure 4B), expression levels of TkBBX1, TkBBX4, TkBBX15, and TkBBX21 were highest at 12 h, while that of TkBBX19 at 6 h. The expression levels of TkBBX7, TkBBX16, and TkBBX17 exhibited a continuous increase. The results were similar to salt treatment, in which the expression levels of TkBBX7 and TkBBX17 genes were strongly up-regulated at 12 h (more than 15-fold).

### 2.6. Subcellular Localization of TkBBX Genes

**Figure 5:**
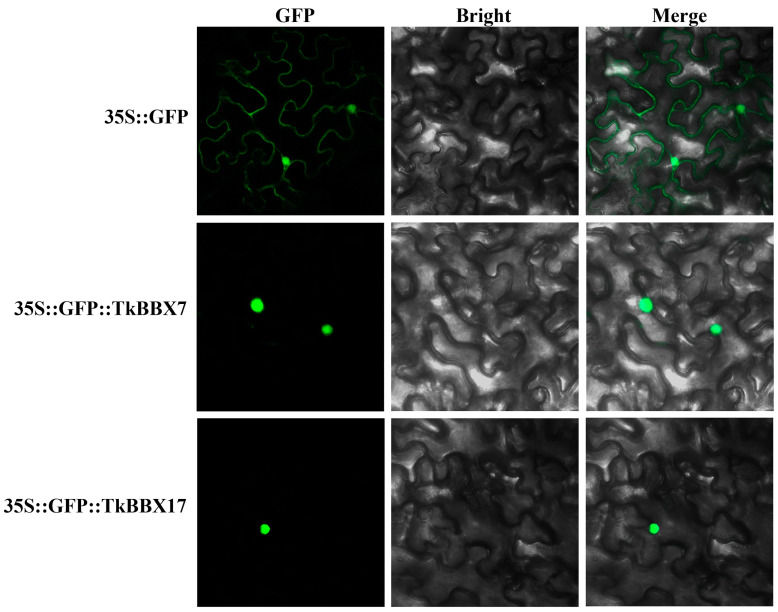
The subcellular localization analysis of TkBBX7 and TkBBX17 proteins.

Subcellular localization provides vital information about a protein’s function. TkBBX7 and TkBBX17 were selected for subcelluar localization experiments. GFP-TkBBX7 and GFP-TkBBX17 plasmids were made and transformed in tobacco. As shown in Figure 5, the green fluorescence of the empty vector (35S::GFP) was distributed on plasma membranes and the nucleus, while GFP-TkBBX7 and GFP-TkBBX17 were exclusively localized in the nucleus. This result was consistent with the website’s predictions and other species’ BBX proteins.

### 2.7. The Tolerance of TkBBXs to Salt and Drought Stresses in Yeast

**Figure 6:**
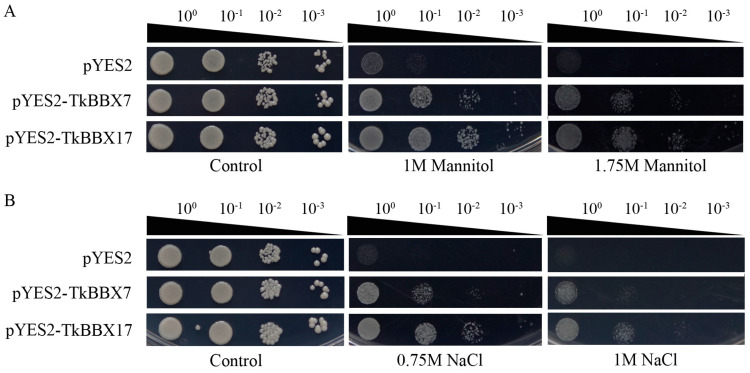
The function analysis of TkBBX genes under salt and drought stresses in yeast strain INVSC1. (**A**): The function analysis of control and TkBBX7 and TkBBX17 genes under different drought stresses in yeast strain INVSC1. (**B**): The function analysis of control and TkBBX7 and TkBBX17 genes under different salt stresses in yeast strain INVSC1.

Through the expression pattern of TkBBX genes under salt and drought treatment, TkBBX7 and TkBBX17, whose expression levels showed a continuous increase, were selected to investigate the biological functions. In order to detect the tolerance of TkBBX7 and TkBBX17 to abiotic stress, drought (1 M, 1.75 M mannitol) and salt (0.75 M, 1 M NaCl) were selected for the experiments (Figure 6). A bacterial solution containing only pYES2-NTB plasmids and SG/−Ura solid medium with no additional NaCl or mannitol added was the negative control. The results indicated that both the control (pYES2−NTB) and the two overexpressing yeast strains showed a consistent growth state on SG−Ura medium, indicating that the overexpression of TkBBXs in the INVSC1 strain had no effect on its growth under normal conditions. Meanwhile, in medium containing 1 M, 1.75 M mannitol (Figure 6A) or 0.75 M, 1 M NaCl (Figure 6B), TkBBX7 and TkBBX17 showed much greater growth than the control. This indicated their potential to enhance salt and drought tolerance in yeast strains.

## 3. Discussion

The BBX gene is a key transcription factor that regulates plant growth, development, and stress response [1,2]. The *BBX* gene family is a kind of zinc finger transcription factor consisting of B-box and CCT domains. It was identified and functionally studied in many plants [4,5,6,7,8,9,10,11,12,13], but not in *T. kirilowii*. In this study, the evolutionary relationships of the TkBBX gene family were analyzed using transcriptome data, and qRT-PCR was used to analyze the gene response to stress treatment. Furthermore, we selected *TkBBX7* and *TkBBX17* for subcellular localization assays and functional analysis.

We identified 25 TkBBX members and divided these proteins into five subfamilies. It is well known that, in Arabidopsis, the BBX family is divided into five subgroups based on the presence and number of B-box domains and CCT protein domains [6]. Both the first and second branches contain two B-box domains and one CCT protein domain. The third branch consists of a B-box domain and a CCT domain, the fourth branch contains two B-box domains, and the fifth branch has only one B-box domain. In this study, a neighbor-joining (NJ) evolutionary tree was constructed and divided into five subgroups based on the *A. thaliana* BBX protein sequence. However, unlike *A. thaliana*, *T. kirilowii* BBX cannot be grouped into subfamilies according to its domain (Figure 2). For each subfamily, TkBBX differs from AtBBX in both the type and number of domains. For example, TkBBX12 and TkBBX19 in subfamily I both contained two B-box domains and one CCT domain, which is the same as pineapple [40] and quinoa [31]. But, the members in *A. thaliana* subfamily I contained only one B-box domain. Previous studies suggested that this may be because some B-box 2 may have been removed during evolution. Researchers discovered significant variations in the gene structure and molecular characteristics of the BBX gene in plants by examining the development and growth of the BBX gene family, indicating that the BBX family is highly diverse [41].

Ka/Ks ratios were calculated to analyze the evolutionary process of the *T. kirilowii* BBX gene family. In general, a Ka/Ks ratio greater than 1 means positive selection for evolutionary acceleration, a Ka/Ks ratio equal to 1 represents neutral selection, and a Ka/KS ratio less than 1 signifies negative selection for evolutionary acceleration. In *T. kirilowii*, Ka/Ks values for all homologous pairs were less than 1, suggesting that these genes have undergone negative purification selection during evolution, and this is consistent with quinoa [31].

BBX genes were identified to be involved in many processes of plant growth and development, such as seedling photomorphogenesis, flowering, plant hormone signal transduction, pigment accumulation, and abiotic and biological stress [42]. Nevertheless, the function of *TkBBXs* has not been studied. BBX genes regulate flowering in herbaceous plants [43,44,45], and *PtCO2* was shown to be associated with poplar growth arrest and flower bud formation [46]. And in *Platanus × acerifolia*, some BBX genes, such as *PaBBX1-1*, *PaBBX4*, *PaBBX5-1/2*, *PaBBX7*, *PaBBX8-1/2*, and *PaBBX11-1*, showed high expressions during the blossoming transition period [47]. These results suggested that the BBX gene may be involved in flowering and dormancy. In our study, some TkBBX genes were highly expressed during bud and fade bloom, for example, the *TkBBX19* gene in female flowers and the TkBBX7 gene in male flowers. In addition, numerous genes, such as *TkBBX12*, *13*, *15*, *16*, and *17* in female flowers and *TkBBX3*, *5*, *15*, *16*, *19*, and *25* in male flowers, were highly expressed in preliminary bloom. This indicated that these TkBBX genes may be involved in the flowering transition.

Because the BBX gene has multiple functions, we were still concerned about the response to abiotic stress. Previous studies reported that, in alfalfa, most MsBBX genes have positive responses to drought or salt stress [48]. And in *T. kirilowii*, a similar pattern was found. Only the *TkBBX19* gene was down-regulated under NaCl treatment, and the *TkBBX3* gene was down-regulated under PEG treatment. A similar situation occurred in other plants. For example, *GmBBX5b*/*15c*/*15d*/*21d/2 1 g/24d/27a/28e/28f* showed an increased expression pattern under salt stress conditions, and only *GmBBX21c*’s expression level was decreased [49]. All of these results suggested that these genes may have potential roles of plants in drought or salt tolerance and were positive regulators of drought and salt stress signaling in *T. kirilowii*. It is well known that salt and drought stress can cause crop growth restriction, affecting yield significantly and causing it to decline [50]. Increasing evidence indicated that BBX genes can affect both plant growth and development and plant response to stress. For example, overexpression of AtSTO (AtBBX24) promoted root growth of *A. thaliana* under high-salinity conditions [34]. The survival rate of MdBBX1 transgenic plants was higher under salt stress [51]. In our study, TkBBX7 was highly expressed not only in male and female flower buds but also in salt and drought treatment. We hypothesize that *TkBBX7* played a role in both growth and development and in response to stress.

After a thorough analysis of all the results, two genes were identified, namely TkBBX7 and TkBBX17. Their expression levels are continuously up-regulated under NaCl and PEG stress conditions. Transgenic yeasts also show some resistance to stress. These findings suggest that the two genes may play a major role in the response of *T. kirilowii* to abiotic stress, and further study of their molecular regulatory mechanisms may help improve the biological and abiotic stress resistance of *T. kirilowii*.

## 4. Materials and Methods

### 4.1. Plant Materials, Growth Conditions, and Stress Treatments

The experimental materials (*T. kirilowii* tissue culture seedlings) used in this study were kept at the Institute of Horticulture, Anhui Province, China. Six-week-old seedlings grown on MS medium were used for relative expression level comparisons of candidate genes under abiotic stress. These seedings were grown under conditions of 16 h light/8 h dark at 22 °C. The leaves for tissue culture were treated with 20% PEG 6000 (Polyethylene Glycol 6000, Sangon, Shanghai, China) and 300 mM NaCl (Shanghai, Sangon) solution, respectively, to simulate drought and salt stress. All the leaves were harvested for 1, 3, 6, 12, 24 h treatments, while 0 h was used as control. Analyses were conducted with three biological and three technical replicates. The samples were frozen in liquid nitrogen and stored at −80 °C for RNA extraction.

### 4.2. Identification of the BBX Gene Family in T. kirilowii

The transcriptome data used in this study were uploaded to the National Center for Biotechnology Information (NCBI) website (https://www.ncbi.nlm.nih.gov/ (accessed on 20 January 2025)) (project accession number PRJNA858494). The *Arabidopsis* Information Resource (http://www.arabidopsis.org) was used to download *Arabidopsis thaliana* B-box protein sequences. The Pfam database (http://pfam.xfam.org/ (accessed on 20 January 2025)) was used to obtain *TkBBX* candidate genes and the complete B-box domain was verified using the NCBI online tool CDD (http://www.ncbi.nlm.nih.gov/Structure/cdd/wrpsb.cgi (accessed on 20 January 2025)). The genes’ amino acid number, open reading frame (ORF) length (bp), isoelectric point (pI), and molecular weight (MW) were calculated on the ExPASy website (https://web.expasy.org/compute_pi/pi_tool-doc.html (accessed on 20 January 2025)) [52]. The WOLF PSORT (http://www.genscript.com/psort.html (accessed on 20 January 2025)) website was used to predict the subcellular localization of the TkBBX proteins [53].

### 4.3. Phylogenetic and Conserved Motif Analysis of TkBBX Gene Family

An unrooted phylogenetic tree was constructed by MEGA 11.0 software using the neighbor-joining method with 1000 replications [54]. The MEME online tool (https://meme-suite.org/meme/ (accessed on 20 January 2025)) was used to identify conserved motifs [55]. For paralogous pairs in *T. kirilowii* and orthologous pairs between *T. kirilowii* and *A. thaliana*, Ka, Ks, and Ka/Ks values for homologous pairs were determined and obtained based on previous research [56].

### 4.4. Expression Pattern Analysis of TkBBX Genes in Different Flower Periods

The fold change (FC) and significant q-values were calculated by TBtools 2.136 software [57]. Those genes that satisfied both FC > 1 and *p* < 0.05 were defined as differentially expressed genes (DEGs). The FPKM values of *TkBBX* were used to draw heat maps.

### 4.5. RNA Extraction and qRT-PCR Analysis

Total RNA of leaves was extracted using the Spin Column Plant Total RNA Purification Kit (Shanghai, Sangon) according to the manufacturer’s instructions. The integrity of RNA was detected by 1% agarose gel electrophoresis, and a NanoDrop 2000 spectrophotometer (ThermoFisher Scientific, Wilmington, DE, USA) was used to detected the concentration of purified RNAs. Total RNA was reverse transcribed into cDNA utilizing UnionScript First-stand cDNA Synthesis Mix (Genesand Biotech Co., Ltd., Beijing, China) and all the samples were stored at −20 °C. The qRT-PCR was performed using GS AntiQ qPCR SYBR Master Mix (Taraka, Beijing, China). The reaction procedure was carried out in an ABI7500 thermal circulator (Applied Bio-systems, Foster City, CA, USA). The relative expression was calculated using the 2^−∆∆CT^ method [58]. GAPDH was used as an internal control [59] and GraphPad 8 software was used for statistical analysis [60]. The primers used in this experiment are listed in Table S1.

### 4.6. Subcellular Localization Assay

The full-length CDSs of TkBBX7 and TkBBX17 genes were cloned from *T. kirilowii*, and the products were inserted into a pMD43 vector containing the CaMV35S promoter and GFP. The recombinant vector plasmid and empty vector plasmid were transformed into Agrobacterium competent GV3101, respectively. The cell suspensions were injected into tobacco leaves for instantaneous transformation. After 48 h, the green fluorescence signal was observed by a laser scanning confocal microscope (LSM 880, Zeiss, Wetzlar, Germany).

### 4.7. Functional Verification of TkBBX7 and TkBBX17 in Yeast

The CDS of *TkBBX7* and *TkBBX17* were inserted into pYES2-NTB vectors to create the fusion plasmids *pYES2*-*TkBBX7* and *pYES2*-*TkBBX17*. The empty vector and two fusion vectors were transformed into the yeast strain INVSC1, respectively. The yeast cell suspensions were coated on SD-Ura medium and cultured at 29 °C for 72–96 h. SD/-Ura liquid medium was used to culture positive colonies until OD600 was just above 1.2, then SG/-Ura liquid medium was used to induce expression. The yeast cell suspensions were diluted to an OD600 of 0.5 and further diluted 10, 100, and 1000 times. The bacterial solutions grown on SG/Ura plates and cultured at 30 °C were the control group. For drought and salt treatment, the continuously diluted bacterial solution was plated on SG/-Ura medium containing mannitol (1.5 M, 1.75 M) and NaCl (0.75 M, 1 M), respectively.

## 5. Conclusions

A total of 25 BBX genes were identified and systematically analyzed in the *T. kirilowii* transcriptome. The evolutionary features of *TkBBX* genes were examined through phylogenetic comparison and homology. Based on the expression levels during flowering and under drought and salt treatment, *TkBBX7* and *TkBBX17* were screened for further experiments. Subcellular localization experiments indicated that the candidate genes have typical transcription factor characteristics, that is, they are all located in the nucleus. Transgenic yeast experiments demonstrated that TkBBX7 and TkBBX17 both enhanced yeast tolerance to salt and drought. In summary, these findings offer a valuable reference for comprehending the distinct biological function of *TkBBX* genes in *T. kirilowii*.

## Figures and Tables

**Table 1:** Characterization of *TkBBXs* identified in *Trichosanthes kirilowi*.

Name	ORF Length (bp)	Protein			Subcellular Localization
		Length (aa)	PI	MW (Da)	
TkBBX1	1251	416	5.34	45,315.65	nucleus
TkBBX2	591	196	5.42	21,528.00	nucleus
TkBBX3	1071	356	4.74	40,194.50	nucleus
TkBBX4	1227	408	5.48	44,870.00	nucleus
TkBBX5	1197	398	6.22	44,811.11	nucleus
TkBBX6	933	310	5.30	34,189.24	nucleus
TkBBX7	834	277	4.24	30,376.77	nucleus
TkBBX8	1251	416	5.27	45,256.58	nucleus
TkBBX9	954	317	4.79	35,435.72	nucleus
TkBBX10	1101	366	4.82	39,790.15	nucleus
TkBBX11	687	228	4.86	25,319.24	nucleus
TkBBX12	954	317	8.22	34,707.13	nucleus
TkBBX13	480	159	6.86	17,469.76	nucleus
TkBBX14	312	103	10.42	11,615.83	nucleus
TkBBX15	888	295	5.36	32,186.03	nucleus
TkBBX16	708	235	5.02	25,915.49	nucleus
TkBBX17	846	281	4.20	31,019.26	nucleus
TkBBX18	651	216	6.37	23,578.75	nucleus
TkBBX19	951	316	7.51	34,439.71	nucleus
TkBBX20	456	151	5.70	16,623.66	nucleus
TkBBX21	1227	408	5.31	44,798.88	nucleus
TkBBX22	228	75	5.13	8068.35	nucleus
TkBBX23	669	222	5.60	24,372.74	nucleus
TkBBX24	462	153	6.74	17,176.36	nucleus
TkBBX25	1323	440	6.12	49,928.00	nucleus

**Table 2:** Ka, Ks, and Ka/Ks values for the BBX genes in Trichosanthes and Arabidopsis.

Paralogous Pairs	Ka	Ks	Ka/Ks
TkBBX1/TkBBX18	0.062811	0.084951	0.73938
TkBBX2/TkBBX4	0.013396	0.022376	0.598669
TkBBX3/TkBBX25	0.027515	0.055871	0.492471
TkBBX5/TkBBX25	0.349804354	3.031300959	0.115397434
TkBBX8/TkBBX18	0.064963628	0.077480506	0.838451254
TkBBX7/TkBBX17	0.007762036	0.010979153	0.706979455
TkBBX9/TkBBX11	0.008541693	0.009793393	0.872189379
TkBBX10/TkBBX21	0.00471746	0.012224379	0.385905915
TkBBX13/TkBBX20	0.017279382	0.06141537	0.281352732
TkBBX22/TkBBX24	0.064695258	0.18555613	0.348656001
**Orthologous pairs**	**Ka**	**Ks**	**Ka/Ks**
TkBBX8/AtBBX7	0.257420338	2.43798484	0.105587342
TkBBX11/AtBBX15	0.3645691		
TkBBX12/AtBBX6	0.373713424	3.080075312	0.12133256
TkBBX18/AtBBX25	0.223789243		
TkBBX18/AtBBX8	0.356460289		
TkBBX20/AtBBX18	0.256192316		
TkBBX23/AtBBX20	0.324254708		
TkBBX24/AtBBX19	0.168224215		
TkBBX25/AtBBX14	0.419126264		

## Data Availability

The raw sequencing data have been deposited in the National Center for Biotechnology Information (NCBI) database under accession number PRJNA858494.

## Supplementary Materials

The following supporting information can be downloaded at: https://www.mdpi.com/article/10.3390/plants14060975/s1, Table S1: The primers used in this study.

## References

[1] Gangappa S.N., Botto J.F. (2014). The BBX family of plant transcription factors. Trends Plant Sci..

[2] Han G., Qiao Z., Li Y., Yang Z., Wang C., Zhang Y., Liu L., Wang B. (2022). RING Zinc Finger Proteins in Plant Abiotic Stress Tolerance. Front. Plant Sci..

[3] Liu Y., Khan A.R., Gan Y. (2022). C2H2 Zinc Finger Proteins Response to Abiotic Stress in Plants. Int. J. Mol. Sci..

[4] Cheng X., Lei S., Li J., Tian B., Li C., Cao J., Lu J., Ma C., Chang C., Zhang H. (2024). In silico analysis of the wheat BBX gene family and identification of candidate genes for seed dormancy and germination. BMC Plant Biol..

[5] Cao J., Yuan J., Zhang Y., Chen C., Zhang B., Shi X., Niu R., Lin F. (2023). Multi-layered roles of BBX proteins in plant growth and development. Stress Biol..

[6] Putterill J., Robson F., Lee K., Simon R., Coupland G. (1995). The CONSTANS gene of arabidopsis promotes flowering and encodes a protein showing similarities to zinc finger transcription factors. Cell.

[7] Hassidim M., Harir Y., Yakir E., Kron I., Green R.M. (2009). Over-expression of CONSTANS-LIKE 5 can induce flowering in short-day grown Arabidopsis. Planta.

[8] Li F., Sun J., Wang D., Bai S., Clarke A.K., Holm M. (2014). The B-box family gene STO (BBX24) in Arabidopsis thaliana regulates flowering time in different pathways. PLoS ONE.

[9] Cheng X.F., Wang Z.Y. (2005). Overexpression of COL9, a CONSTANS-LIKE gene, delays flowering by reducing expression of CO and FT in Arabidopsis thaliana. Plant J..

[10] Tripathi P., Carvallo M., Hamilton E.E., Preuss S., Kay S.A. (2017). Arabidopsis B-BOX32 interacts with CONSTANS-LIKE3 to regulate flowering. Proc. Natl. Acad. Sci. USA.

[11] Wang L., Sun J., Ren L., Zhou M., Han X., Ding L., Zhang F., Guan Z., Fang W., Chen S. (2020). CmBBX8 accelerates flowering by targeting CmFTL1 directly in summer chrysanthemum. Plant Biotechnol. J..

[12] Bai B., Zhao J., Li Y., Zhang F., Zhou J., Chen F., Xie X. (2016). OsBBX14 delays heading date by repressing florigen gene expression under long and short-day conditions in rice. Plant Sci..

[13] Liu H., Dong S., Sun D., Liu W., Gu F., Liu Y., Guo T., Wang H., Wang J., Chen Z. (2016). CONSTANS-Like 9 (OsCOL9) Interacts with Receptor for Activated C-Kinase 1(OsRACK1) to Regulate Blast Resistance through Salicylic Acid and Ethylene Signaling Pathways. PLoS ONE.

[14] Park H.J., Kim W.Y., Pardo J.M., Yun D.J. (2016). Molecular Interactions Between Flowering Time and Abiotic Stress Pathways. Int. Rev. Cell Mol. Biol..

[15] Datta S., Hettiarachchi C., Johansson H., Holm M. (2007). SALT TOLERANCE HOMOLOG2, a B-box protein in Arabidopsis that activates transcription and positively regulates light-mediated development. Plant Cell.

[16] Datta S., Johansson H., Hettiarachchi C., Irigoyen M.L., Desai M., Rubio V., Holm M. (2008). LZF1/SALT TOLERANCE HOMOLOG3, an Arabidopsis B-box protein involved in light-dependent development and gene expression, undergoes COP1-mediated ubiquitination. Plant Cell.

[17] Datta S., Hettiarachchi G.H., Deng X.W., Holm M. (2006). Arabidopsis CONSTANS-LIKE3 is a positive regulator of red light signaling and root growth. Plant Cell.

[18] Chang C.S., Li Y.H., Chen L.T., Chen W.C., Hsieh W.P., Shin J., Jane W.N., Chou S.J., Choi G., Hu J.M. (2008). LZF1, a HY5-regulated transcriptional factor, functions in Arabidopsis de-etiolation. Plant J..

[19] Fan X.Y., Sun Y., Cao D.M., Bai M.Y., Luo X.M., Yang H.J., Wei C.Q., Zhu S.W., Sun Y., Chong K. (2012). BZS1, a B-box protein, promotes photomorphogenesis downstream of both brassinosteroid and light signaling pathways. Mol. Plant.

[20] Gangappa S.N., Crocco C.D., Johansson H., Datta S., Hettiarachchi C., Holm M., Botto J.F. (2013). The Arabidopsis B-BOX protein BBX25 interacts with HY5, negatively regulating BBX22 expression to suppress seedling photomorphogenesis. Plant Cell.

[21] Holtan H.E., Bandong S., Marion C.M., Adam L., Tiwari S., Shen Y., Maloof J.N., Maszle D.R., Ohto M.A., Preuss S. (2011). BBX32, an Arabidopsis B-Box protein, functions in light signaling by suppressing HY5-regulated gene expression and interacting with STH2/BBX21. Plant Physiol..

[22] Indorf M., Cordero J., Neuhaus G., Rodríguez-Franco M. (2007). Salt tolerance (SO), a stress-related protein, has a major role in light signalling. Plant J..

[23] Kumagai T., Ito S., Nakamichi N., Niwa Y., Murakami M., Yamashino T., Mizuno T. (2008). The common function of a novel subfamily of B-Box zinc finger proteins with reference to circadian-associated events in Arabidopsis thaliana. Biosci. Biotechnol. Biochem..

[24] Khanna R., Shen Y., Toledo-Ortiz G., Kikis E.A., Johannesson H., Hwang Y.S., Quail P.H. (2006). Functional profiling reveals that only a small number of phytochrome-regulated early-response genes in Arabidopsis are necessary for optimal deetiolation. Plant Cell.

[25] An J.P., Wang X.F., Zhang X.W., Bi S.Q., You C.X., Hao Y.J. (2019). MdBBX22 regulates UV-B-induced anthocyanin biosynthesis through regulating the function of MdHY5 and is targeted by MdBT2 for 26S proteasome-mediated degradation. Plant Biotechnol. J..

[26] Fang H., Dong Y., Yue X., Hu J., Jiang S., Xu H., Wang Y., Su M., Zhang J., Zhang Z. (2019). The B-box zinc finger protein MdBBX20 integrates anthocyanin accumulation in response to ultraviolet radiation and low temperature. Plant Cell Environ..

[27] Crocco C.D., Holm M., Yanovsky M.J., Botto J.F. (2011). Function of B-BOX under shade. Plant Signal Behav..

[28] Crocco C.D., Holm M., Yanovsky M.J., Botto J.F. (2010). AtBBX21 and COP1 genetically interact in the regulation of shade avoidance. Plant J..

[29] An J.P., Wang X.F., Espley R.V., Lin-Wang K., Bi S.Q., You C.X., Hao Y.J. (2020). An Apple B-Box Protein MdBBX37 Modulates Anthocyanin Biosynthesis and Hypocotyl Elongation Synergistically with MdMYBs and MdHY5. Plant Cell Physiol..

[30] Liu Y., Zhang X.W., Liu X., Zheng P.F., Su L., Wang G.L., Wang X.F., Li Y.Y., You C.X., An J.P. (2022). Phytochrome interacting factor MdPIF7 modulates anthocyanin biosynthesis and hypocotyl growth in apple. Plant Physiol..

[31] Xuefen D., Wei X., Wang B., Xiaolin Z., Xian W., Jincheng L. (2022). Genome-wide identification and expression pattern analysis of quinoa BBX family. PeerJ.

[32] Bai S., Saito T., Honda C., Hatsuyama Y., Ito A., Moriguchi T. (2014). An apple B-box protein, MdCOL11, is involved in UV-B- and temperature-induced anthocyanin biosynthesis. Planta.

[33] Bai S., Tao R., Tang Y., Yin L., Ma Y., Ni J., Yan X., Yang Q., Wu Z., Zeng Y. (2019). BBX16, a B-box protein, positively regulates light-induced anthocyanin accumulation by activating MYB10 in red pear. Plant Biotechnol J..

[34] Nagaoka S., Takano T. (2003). Salt tolerance-related protein STO binds to a Myb transcription factor homologue and confers salt tolerance in Arabidopsis. J. Exp. Bot..

[35] Liu X., Li R., Dai Y., Yuan L., Sun Q., Zhang S., Wang X. (2019). A B-box zinc finger protein, MdBBX10, enhanced salt and drought stresses tolerance in Arabidopsis. Plant Mol. Biol..

[36] Huang S., Chen C., Xu M., Wang G., Xu L.A., Wu Y. (2021). Overexpression of Ginkgo BBX25 enhances salt tolerance in Transgenic Populus. Plant Physiol. Biochem..

[37] Wu H., Wang X., Cao Y., Zhang H., Hua R., Liu H., Sui S. (2021). CpBBX19, a B-Box Transcription Factor Gene of Chimonanthus praecox, Improves Salt and Drought Tolerance in Arabidopsis. Genes.

[38] Liu Y., Chen H., Ping Q., Zhang Z., Guan Z., Fang W., Chen S., Chen F., Jiang J., Zhang F. (2019). The heterologous expression of CmBBX22 delays leaf senescence and improves drought tolerance in Arabidopsis. Plant Cell Rep..

[39] Wang Q., Tu X., Zhang J., Chen X., Rao L. (2013). Heat stress-induced BBX18 negatively regulates the thermotolerance in Arabidopsis. Mol. Biol. Rep..

[40] Ouyang Y., Pan X., Wei Y., Wang J., Xu X., He Y., Zhang X., Li Z., Zhang H. (2022). Genome-wide identification and characterization of the BBX gene family in pineapple reveals that candidate genes are involved in floral induction and flowering. Genomics.

[41] Yu L., Lyu Z., Liu H., Zhang G., He C., Zhang J. (2022). Insights into the evolutionary origin and expansion of the BBX gene family. Plant Biotechnol Rep..

[42] Song Z., Bian Y., Liu J., Sun Y., Xu D. (2020). B-box proteins: Pivotal players in light-mediated development in plants. J. Integr. Plant Biol..

[43] Talar U., Kiełbowicz-Matuk A. (2021). Beyond Arabidopsis: BBX Regulators in Crop Plants. Int. J. Mol. Sci..

[44] Susila H., Nasim Z., Gawarecka K., Jung J.Y., Jin S., Youn G., Ahn J.H. (2023). Chloroplasts prevent precocious flowering through a GOLDEN2-LIKE-B-BOX DOMAIN PROTEIN module. Plant Commun..

[45] Xu X., Xu J., Yuan C., Chen Q., Liu Q., Wang X., Qin C. (2022). BBX17 Interacts with CO and Negatively Regulates Flowering Time in Arabidopsis thaliana. Plant Cell Physiol..

[46] Böhlenius H., Huang T., Charbonnel-Campaa L., Brunner A.M., Jansson S., Strauss S.H., Nilsson O. (2006). CO/FT regulatory module controls timing of flowering and seasonal growth cessation in trees. Science.

[47] Shi G., Ai K., Yan X., Zhou Z., Cai F., Bao M., Zhang J. (2023). Genome-Wide Analysis of the *BBX* Genes in *Platanus* × *acerifolia* and Their Relationship with Flowering and/or Dormancy. Int. J. Mol. Sci..

[48] Li S., Guo S., Gao X., Wang X., Liu Y., Wang J., Li X., Zhang J., Fu B. (2024). Genome-wide identification of B-box zinc finger (BBX) gene family in Medicago sativa and their roles in abiotic stress responses. BMC Genom..

[49] Shan B., Bao G., Shi T., Zhai L., Bian S., Li X. (2022). Genome-wide identification of BBX gene family and their expression patterns under salt stress in soybean. BMC Genom..

[50] Papiernik S.K., Grieve C.M., Lesch S.M., Yates S.R. (2005). Effects of salinity, imazethapyr, and chlorimuron application on soybean growth and yield. Commun. Soil Sci. Plant Anal..

[51] Dai Y.Q., Lu Y., Zhou Z., Wang X.Y., Ge H.J., Sun Q.H. (2022). B-box containing protein 1 from Malus domestica (MdBBX1) is involved in the abiotic stress response. Peerj..

[52] Artimo P., Jonnalagedda M., Arnold K., Baratin D., Csardi G., de Castro E., Duvaud S., Flegel V., Fortier A., Gasteiger E. (2012). ExPASy: SIB bioinformatics resource portal. Nucleic Acids Res..

[53] Horton P., Park K.J., Obayashi T., Fujita N., Harada H., Adamscollier C.J., Nakai K. (2007). WoLF PSORT: Protein localization predictor. Nucleic Acids Res..

[54] Tamura K., Stecher G., Kumar S. (2021). MEGA11: Molecular evolutionary genetics analysis version 11. Mol. Biol. Evol..

[55] Bailey T.L., Boden M., Buske F.A., Frith M., Grant C.E., Clementi L., Ren J., Li W.W., Noble W.S. (2009). MEME SUITE: Tools for motif discovery and searching. Nucleic Acids Res..

[56] Xiong R., Chu Z., Peng X., Cui G., Li W., Dong L. (2023). Transcript-wide identification and expression pattern analysis to comprehend the roles of AP2/ERF genes under development and abiotic stress in *Trichosanthes kirilowii*. BMC Plant Biol..

[57] Chen C., Chen H., Zhang Y., Thomas H.R., Frank M.H., He Y., Xia R. (2020). TBtools: An Integrative Toolkit Developed for Interactive Analyses of Big Biological Data. Mol. Plant.

[58] Schmittgen T.D., Livak K.J. (2008). Analyzing real-time PCR data by the comparative C(T) method. Nat. Protoc..

[59] Li D., He Y., Li S., Shi S., Li L., Liu Y., Chen H. (2021). Genome-wide characterization and expression analysis of AP2/ERF genes in eggplant (*Solanum melongena* L.). Plant Physiol. Biochem..

[60] Mitteer D.R., Greer B.D. (2022). Using GraphPad Prism’s Heat Maps for Efficient, Fine-Grained Analyses of Single-Case Data. Behav. Anal. Pract..

