# Supplementary material for: Transcript-Wide Identification and Characterization of the BBX Gene Family in Trichosanthes kirilowii and Its Potential Roles in Development and Abiotic Stress
Source: Plants (Basel). 2025 Mar 20;14(6):975. doi: 10.3390/plants14060975 (PMC11946252; doi:10.3390/plants14060975)
Supplement: Supplementary file 1 [file plants-14-00975-s001.zip › plants-3487841-supplementary.pdf]

Table S1.

Name	Primers
TkBBX1F	GCGAGAGTAGTGCTGGTGATTATC
TkBBX1R	ATTGTTACGGTCAGAAGATGGGAAG
TkBBX4F	CAGGAGAACATCAAGACTGTGGAG
TkBBX4R	CGCATTACGGCATCGCTACG
TkBBX5F	AGAAGCGGCGGACGAGATTG
TkBBX5R	CTCTTGACGAACCTCCCTTTCATTC
TkBBX7F	TGACGGAGATGATAGCGAGGAAG
TkBBX7R	AAGACCACGGCACCACCTG
TkBBX15F	GTTGAACTGGACGACGAAGATGAG
TkBBX15R	GCTGTGGGTGGAGAGGGAATC
TkBBX16F	GCTGCTGCTGAAGTTCCTGAG
TkBBX16R	TCCTCATCATCTACCACCTCAATCC
TkBBX17F	AGACGATGACGAGGACGATGAC
TkBBX17R	AAGACCACGGCACCACCTG
TkBBX19F	CCACGACGCCGCCTTCC
TkBBX19R	CAGACCTCGCAGAGCCAGAC
TkBBX21F	CAGGAGAACATCAAGACTGTGGAG
TkBBX21R	CGCATTACGGCATCGCTACG
pYES2-TkBBX7F	cgacgatgacgataaggtaccTATGAAGAAGTGCGAGCTGTGTGGG
pYES2-TkBBX7R	gaattccaccacactggatccTTAACGATTCTGATCTCTGCTCAT
pYES2-TkBBX17F	cgacgatgacgataaggtaccTATGAAGATTCAGTGCAATGTCTGCGAGA
pYES2-TkBBX17R	gaattccaccacactggatccTTAGAACTGCCTCCTTCGTTTCG
pMD43-TkBBX7F	ATGGATGAACTATACAAAGGGATGAAGAAGTGCGAGCTGTGTGGG
pMD43-TkBBX7R	AACATATCCAGTCACTATGGGTTAACGATTCTGATCTCTGCTCAT
pMD43-TkBBX17F	ATGGATGAACTATACAAAGGGATGAAGATTCAGTGCAATGTCTGCGAGA
pMD43-TkBBX17R	AACATATCCAGTCACTATGGGTTAGAACTGCCTCCTTCGTTTCG
